# 

**Published:** 1988-05

**Authors:** 


Copyright © Clinical Press Ltd 1988

### Contents

3 **An Introduction to Magnetic Resonance Imaging**
Terry Beddoe and Paul Goddard
5 **The Bristol MRI Centre**
Gordon Thomson
7 **The Physics of Magnetic Resonance Imaging**
Paul Goddard and Peter Jackson
8 **MRI Pulse Sequences**
P. Goddard
9 **MRI and the District General Hospital**
C. Johnson and J. B. Penry
12 **MRI in Pregnancy**
M. J. Nimmo, D. Kinsella and H. S. Andrews
13 **Magnetic Resonance Imaging of the CNS**
J. R. Bradshaw and T. T. Lewis
19 **Magnetic Resonance Imaging of the Orbit**
A. Berger, T. T. Lewis and P. Goddard
21 **Magnetic Resonance Imaging in the Investigation of Temporo-Mandibular Joint Disorders**
M. H. Morse
23 **Cardiovascular MRI**
G. Hartnell
25 **Magnetic Resonance Imaging of the Pancreas**
J. Kabala, J. Virjee, R. Mountford, J. Waring, R. Yeats and P. Goddard
26 **MRI in Oncology**
P. Goddard
26 **The Stir Sequence in MRI of Neoplastic Lesions**
P. Goddard, J. Waring, A. Case, R. Yeats, J. A. Bullimore, E. Whipp and V. Barley
27 **MRI in Thoracic Malignancy**
P. Goddard
29 **The Detection of Metastatic Disease of the Spine in Oncology Patients using MRI**
P. Goddard, I. Watt, E. R. Davies, P. Cook, J. Waring and B. Hale
32 **The Detection of Recurrent and Metastatic Malignant Disease in the Pelvis using MRI**
P. Goddard, W. Wong, R. Yeats, A. Case, J. Tawn, J. Browning and E. Whipp
34 **Gadolinium—DPTA**
H. P. Niendorf
35 **Magnetic Resonance Imaging Systems Equipment Hardware Today and Tomorrow**
D. C. Beatty

